# PACAP38 synergizes with irradiation to suppress the proliferation of multiple cancer cells via regulating SOX6/Wnt/β-catenin signaling

**DOI:** 10.3389/fphar.2024.1492453

**Published:** 2024-10-22

**Authors:** Ran Wu, Chun-Xiang Cao, Lu Cao, Jun Su, Ke-Man Liao, Huan Li, Qian Zhu, Shu-Yan Li, Min Li, Jia-Yi Chen

**Affiliations:** ^1^ Department of Radiation Oncology, Ruijin Hospital, Shanghai Jiaotong University School of Medicine, Shanghai, China; ^2^ Shanghai Key Laboratory of Proton-Therapy, Shanghai, China; ^3^ Department of Radiation Oncology, Shanghai 10th People’s Hospital, Tongji University School of Medicine, Shanghai, China; ^4^ Department of Oncology, Xin-Hua Hospital, Shanghai Jiaotong University School of Medicine, Shanghai, China

**Keywords:** PACAP38, cancer, radiotherapy, Sox6, Wnt/β-catenin, histone

## Abstract

**Background:**

Pituitary adenylate cyclase-activating polypeptide **(**PACAP) 38 is an endogenous neuropeptide with diverse functions, notably its critical role in inhibiting tumor proliferation. Radiotherapy is an important step in the standard treatment modality of many tumors. Combining radiotherapy with therapeutic agents represents a new and promising trend aimed at enhancing radiation sensitivity and improving tumor treatment efficacy. However, the efficacy of PACAP38 combined with radiotherapy on tumors has not yet been studied.

**Objective:**

This study aimed to investigate the impact of PACAP38, both independently and in combination with irradiation, on glioma and breast cancer cells, while elucidating the underlying mechanisms involved.

**Methods:**

We investigated the impact of PACAP38 independently and combined it with irradiation on glioma and breast cancer cells *in vitro* through cell counting kit-8, clonogenic formation, Edu assays, and *in vivo* through a xenograft tumor model. We further explored the molecular mechanisms underlying the inhibitory effects of PACAP38 on tumors using RNA sequencing, western blotting assay, immunohistochemistry, and immunofluorescence analysis. Further investigation of gene function and the downstream mechanism was carried out through small interfering RNA and overexpression lentivirus targeting the SRY-related high-mobility group box 6 (SOX6) gene and western blotting assay.

**Results:**

Our findings revealed that PACAP38 could effectively synergize with radiation to suppress the proliferation of glioma and breast cancer cells *in vivo* and *in vitro*. Molecular studies revealed that the inhibitory effect of PACAP38 on tumor cell proliferation was mediated by upregulating SOX6 protein expression through histone acetylation, thereby inhibiting the Wnt-β-catenin signaling pathway.

**Conclusion:**

PACAP38 synergizes with irradiation to suppress the proliferation of multiple cancer cells via regulating SOX6/Wnt/β-catenin signaling. This combination may represent a promising therapeutic strategy for cancer treatment, potentially improving outcomes for patients undergoing radiotherapy.

## 1 Introduction

Pituitary adenylate cyclase-activating polypeptide (PACAP), encoded by the gene Adcyap1, is a member of the secretin/glucagon/vasoactive intestinal polypeptide (VIP)/PACAP/growth-hormone-releasing hormone (GHRH) superfamily. PACAP exists in two bioactive forms: PACAP27 and PACAP38 (Arimura et al., 1992). PACAP has three distinct receptors: VPAC1, VPAC2, and PAC1 receptors, which have various splice variants (Harmar et al., 2012; Spengler et al., 1993). Over the past several decades, PACAP38 was known to exert a wide range of functions within the organism, particularly in the realms of alleviating inflammation reactions, promoting tissue repair, and modulating tumorigenesis (Horvath et al., 2019; Szabadfi et al., 2014; Heimesaat et al., 2014; Toth et al., 2020). Previous studies using a mouse model have demonstrated that PACAP38 may exhibit a certain preventative effect on acute radiation-induced myocardial injury, a common side effect in breast cancer patients undergoing radiotherapy (Li et al., 2019).

Malignant tumors pose a significant threat to human health. Glioblastoma (GBM) is the most common primary malignant tumor in the adult central nervous system (Ostrom et al., 2018). The standard treatment primarily involves surgery, followed by concurrent radiotherapy and chemotherapy, and adjuvant chemotherapy. However, the prognosis for patients is extremely poor, with a 5-year survival rate of only 5.6% (Dirven et al., 2014). Breast cancer now ranks as the most prevalent neoplastic disease among women, accounting for an extra 2.3 million cases comprising 11.7% of total cancer occurrences (Sung et al., 2021). Furthermore, breast cancer mortality is at the forefront among malignant tumors in women.

Radiotherapy is a key component in various cancer treatment regimens and can substantially enhance therapeutic efficacy (Arimura et al., 1992; Speers et al., 2015; McGale et al., 2011; Denes et al., 2019). However, due to the heterogeneity of tumors, patients with the same type of cancer often exhibit varying degrees of sensitivity to radiotherapy, with some showing limited responsiveness (Speers et al., 2015). The combination of radiotherapy and pharmacological agents to enhance radiosensitivity and improve treatment outcomes represents a promising new therapeutic trend (Montazersaheb et al., 2024; Zhai et al., 2022; Meattini et al., 2024; Beddok et al., 2021). Nonetheless, due to the heterogeneity of cancer and the variability in individual responses, current strategies combining radiotherapy with other treatments face inherent limitations, including safety concerns and the risk of adverse reactions from combination therapies. Thus, the identification of novel therapeutic strategies and well-defined therapeutic targets is of paramount importance. Several previous studies have demonstrated the tumor-suppressive effects of PACAP38 in a variety of cancers, including breast cancer and gliomas, although studies on the downstream mechanisms of tumor suppression by PACAP38 are far from sufficient (D'Amico et al., 2013; Cochaud et al., 2015; Maugeri et al., 2016). Moreover, the impact of PACAP38 combining with radiotherapy on cancer treatment efficacy also remains unclear.

According to currently understanding, after interacting with these 3 G protein-coupled receptors, PACAP38 can trigger the initiation of the cAMP-protein kinases A (PKA)/protein kinases C (PKC) pathway, subsequently modulating downstream transcription factors (TFs) and cell cycle proteins (Spengler et al., 1993; Georg et al., 2016; Juhász et al., 2015). TFs play a crucial biological role in cancer. The core TFs that serve as regulators of oncogenes or tumor suppressors are currently being explored as prospective therapeutic targets for cancer (Kehl et al., 2017; Arthur et al., 2017; Narayanan et al., 2019). Multiple studies have indicated that PACAP can influence the expression of SRY-related high-mobility group box (SOX) transcription family proteins, including SOX5, SOX6 and SOX9, thereby regulating the development of diseases such as osteogenesis and chondrogenesis (Reglodi et al., 2018; Szegeczki et al., 2020; Szegeczki et al., 2019). The SOX transcription factor families are commonly recognized to modulate cell fate in major lineages (Liang et al., 2020). Previous studies have shown that SOX6 is downregulated and serves as a tumor growth inhibitor in many cancers, including breast cancer, lung adenocarcinoma, osteosarcoma, prostate cancer, and esophageal cancer (Chen et al., 2020; Wang et al., 2016; Zhang et al., 2021). Multiple studies have demonstrated that SOX6 exerts its tumor-suppressive role by downregulating the Wnt/β-catenin signaling pathway (Chen et al., 2020; Zeng and Chen, 2023; Dong et al., 2018), which plays a pivotal role in the initiation and progression of various cancers (Zhang et al., 2016; Han et al., 2017; Valenta et al., 2012; Kahn, 2014).

In this investigation, our objective was to evaluate the influence of PACAP38, either independently or in conjunction with irradiation, on glioma and breast cancer cells. Our findings demonstrated that the individual application of PACAP38 exhibits an inhibitory effect on the proliferation activity of glioma and breast cancer cells. Moreover, when PACAP38 is administered concurrently with irradiation, it demonstrates a synergistic effect in suppressing the proliferation activity of glioma and breast cancer cells. We further employed transcriptomics approaches to elucidate the molecular mechanism underlying the inhibitory impact. Our research suggests that when applied alone or in combination with irradiation, PACAP38 can upregulate the expression of SOX6 by acetylating histones and suppressing the Wnt-β-catenin signaling to exert inhibitory impact on glioma and breast cancer cells.

## 2 Materials and methods

### 2.1 Cell culture

The tumor cells were cultured as previously described (Zhu et al., 2021). This research employed human breast cancer cells (T47D, MCF-7, BT-549, MDA-MB-231), glioma cell line (T98G), and human mammary epithelial cells (MCF-10A) sourced from the Chinese Academy of Sciences, Institute of Biochemistry and Cell Biology (Shanghai, China). All cells were cultured in RPMI 1640 medium (Gibco, United States) or Dulbecco’s modified Eagle’s medium (DMEM, Gibco, United States) supplemented with 10% fetal bovine serum (FBS, Gibco, United States).

PACAP38 and PACAP6-38 were purchased from MedChemExpress (MCE, United States). PACAP38 was solubilized in sterile water to reach 1 mg/mL, aliquoted, and kept at −80°C for further utilization. DNA Methylation Inhibitors (5-Azacytidine, 5Aza), Histone Acetylation Inhibitors (HATi, Anacardic acid, ANA), and Histone Deacetylation Inhibitors (HDACi, Vorinostat, SAHA) were purchased from Selleck Chemicals (Selleck, United States).

### 2.2 Western blot

Protein expression was assessed by Western blotting. Cells were lysed using RIPA buffer (Beyotime, China) supplemented with protease and phosphatase inhibitor-supplied for protein extraction. The BCA protein assay kit (Beyotime, China) was utilized to quantify proteins. Protein was loaded into 10% SDS-polyacrylamide gel electrophoresis (SDS-PAGE) in equivalent amounts for electrophoresis, followed by being transferred to PVDF membranes (Millipore, United States). After transfer, the PVDF membranes was first blocked with 5% no-fat milk for 1 h, followed by incubated with primary antibodies overnight at 4°C. At last, they were further incubated with HRP-conjugated secondary antibodies at room temperature (RT) for 1 h. The primary antibodies listed below were employed: anti-SOX6 (Proteintech, United States), anti-β-catenin (CST, United States), anti-LEF1 (CST, United States), anti-TCF1/TCF7 (CST, United States), anti-MMP7 (CST, United States), anti-cJUN (CST, United States), anti-c-myc (CST, United States), anti-CD44 (CST, United States), anti-Tubulin (CST, United States), anti-GAPDH (CST, UUnited States). The Acetyl-Histone Antibody Sampler Kit (CST, United States) was used to evaluate the acetylation states of histones H2A, H2B, H3, and H4. The dilution ratios for all primary antibodies utilized ranged from 1:500 to 1:1000. Secondary antibodies goat anti-rabbit-HRP (CST, United States) and goat anti-mouse-HRP (CST, United States), both used at 1:3000. ECL system (GE Healthcare, Little Chalfont, United Kingdom) was used for visualizing signals.

### 2.3 Cell proliferation assay and cell cycle analysis

The Cell Counting Kit-8 (CCK8) and colony formation assay were used to measure cell proliferation. The CCK8 test was conducted using CCK8 assay kits (Dojindo, Japan). Briefly, 96-well plates were seeded with 2 × 10^3^ cells, and the absorbance at 450 nm was recorded.

For colony formation assay, cells were plated in 6-well plates and subjected to several treatments based on their assigned group. Following an additional 10 days of cultivation, cells were then fixed in 4% paraformaldehyde and stained with 0.5% crystal violet for further visualizing colony formation.

For Edu assay, cells were plated in 24-well plates, EdU solvent was added to the medium for the cells to ingest and staining and assaying were performed 2 h later.

Flow cytometry was used for the analysis of the cell cycle change. The detailed operational procedures are elucidated in our previous article (Li et al., 2019).

### 2.4 Animal experiment

Experiments and procedures were carried out with the consent of the Animal Ethic Committee of Shanghai Ruijin Hospital (the Ethical Clearance number is SYXK 018-0027). 5 × 10^6^ T47D cells diluted with 200 µL phosphate-buffered saline (PBS) were injected subcutaneously in the proximal right hind limb of female nude BALB/c mice aged 5–6 weeks old.

When the longest diameter of the tumors in tumor-bearing mice reached approximately 5–6 mm, a total of four cohorts of five mice each were utilized: the control group (Ctrl), PACAP38 group (PACAP38), irradiation group (IR), and the combined irradiation and PACAP38 treatment group (PACAP38+IR). A dose of 10 Gy X-ray irradiation was applied to the tumor sites of the mice in the IR and PACAP38+IR groups. The mice in PACAP38, PACAP38+IR groups were administered intraperitoneal injection of PACAP38 (10 µg/100 µL) diluted in 0.9% normal saline solution administration for three consecutive days (the day before irradiation, the day of irradiation, the day following irradiation). Mice in the control group and the irradiation group were given equal volumes of 0.9% saline solution. Tumor sizes in all groups were recorded and subsequently monitored every other day from the day of IR. The formula for calculating tumor volume was 0.5 × length × width^2^. When the maximum tumor volume of approximately 1,000 mm³ in the mice was observed, euthanasia was performed, and tumor specimens were dissected, photographed, and fixed to enable further analysis.

### 2.5 Cell and tumor irradiation

A medical linear accelerator (Varian Trilogy, FL, United States) was used for irradiation with an X-ray beam energy of 6 MV and a dose rate of 300 cGy/min. The external beam irradiation treatment plan and positioning strategy are detailed in our previous study (Li et al., 2019). For *in vivo* irradiation of tumors, nude mice bearing a xenograft of breast cancer cells were intraperitoneally administered pentobarbital 0.2% at a dose of 60 mg/kg for anesthesia. Subsequently, X-rays were locally delivered to the xenograft site.

### 2.6 RNA-seq and data analysis

Following a 12-h treatment of T47D cells with RPMI 1640 complete medium containing 100 nM PACAP38 or an equivalent volume of sterile water for control, cellular total RNA was extracted. The RNA was then further enriched with polyA + by Dynabeads Oligo (dT) magnetic beads. The polyA + RNA was prepared as the input material for library preparations using a NEBNext^®^ Ultra™ II mRNA Library Prep Kit for Illumina^®^ (NEB, United States). Following this HaploX Medical Laboratory Co. Ltd. China carried out paired-end sequencing on an Illumina PE150, with the abundance of gene expression being displayed using fragments per Kilobase of Transcript per Million mapped reads (FPKM).

### 2.7 Small interfering RNA (siRNA) transfection

Three pairs of SOX6-siRNAs (si-SOX6 1#, si-SOX6 2#, si-SOX6 3#) and their negative control siRNAs (si-NC) sequences were designed and manufactured by Genomeditech (Shanghai, China). The siRNA sequences are shown in Supplementary Table S1. For more on siRNA knockdown operations, please see our prior publication (Su et al., 2020). Western blotting was used to determine the knock-down efficiency. The pair of siRNAs with the highest knock-down efficiency was used for subsequent experiments.

### 2.8 Lentivirus packaging and infection

Lentiviral vectors were amplified using DH5α, and HEK293T cells were used for lentiviral packaging. Plasmids were transfected into cells with Polyethylenimine (PEI). The culture supernatant were collected at 48 and 72 h post-transfection, centrifuged, filtered, and concentrated, then infect the target cells, as previously described (Zhu et al., 2021).

### 2.9 Immunohistochemical (IHC) analysis

For cell IHC analysis, cells were cultured in 6-well plates for 48 h. Then fix the cells on ice with pre-cooled acetone for 15 min. 0.5% Triton X-100 solution was used for immersing cells for 15 min in RT and were incubated with 3% H2O2 for 15 min and blocked for 1 h with 2% BSA in RT. Next, the cells were successively incubated with primary antibodies for SOX6 (1:300, Proteintech, United States), secondary antibody (Abcam, United States), and tertiary antibody (SAB complex) for 12 h, 30 min, and 30 min respectively at 37°C. Under the condition of light-avoidance, the cells were observed under the microscope to react with DAB to brown color for about 5 min. Then after counterstaining with hematoxylin for 1 min, Dehydration was carried out using graded alcohols, and the clearing was carried out with xylene. The rate of positive protein expression in cells was immediately examined using a microscope (Olympus BX51).

### 2.10 Immunofluorescence analysis

Cells were cultured in 6-well plates for 48 h before the IF experiment (Zhu et al., 2021). The detailed experimental procedures can be found in our previous study. The primary antibody dilutions SOX6 (1:300, Proteintech, United States) were used. The fluorescence intensity was evaluated using fluorescence microscopy (Olympus BX51) and quantified by ImageJ.

### 2.11 Tissue microarray

The tissue microarray, comprising 131 invasive breast cancer tissue specimens sourced from patients, was obtained from Shanghai Outdo Biotech. The development and application of this tissue microarray product received ethical approval from the Ethics Committee of Shanghai Outdo Biotech.

### 2.12 Statistical analysis

Statistical analyses were conducted utilizing GraphPad Prism9 (GraphPad Software). Data were presented as means with standard deviation (SD). The comparison between the two groups employed the unpaired Student’s t-test, while multiple group comparisons were assessed through one‐way analysis of variance (ANOVA). Survival analysis was performed using the Kaplan-Meier method, and significance in survival discrepancies was determined by log-rank tests. A stringent significance threshold was employed in this study, and a two-tailed test with a *p*-value below 0.05 was considered statistically significant. Graphs were generated using GraphPad Prism9 software for comprehensive data visualization.

## 3 Results

### 3.1 PACAP38 suppressed the proliferation ability of glioma and breast cancer cells

**FIGURE 1:**
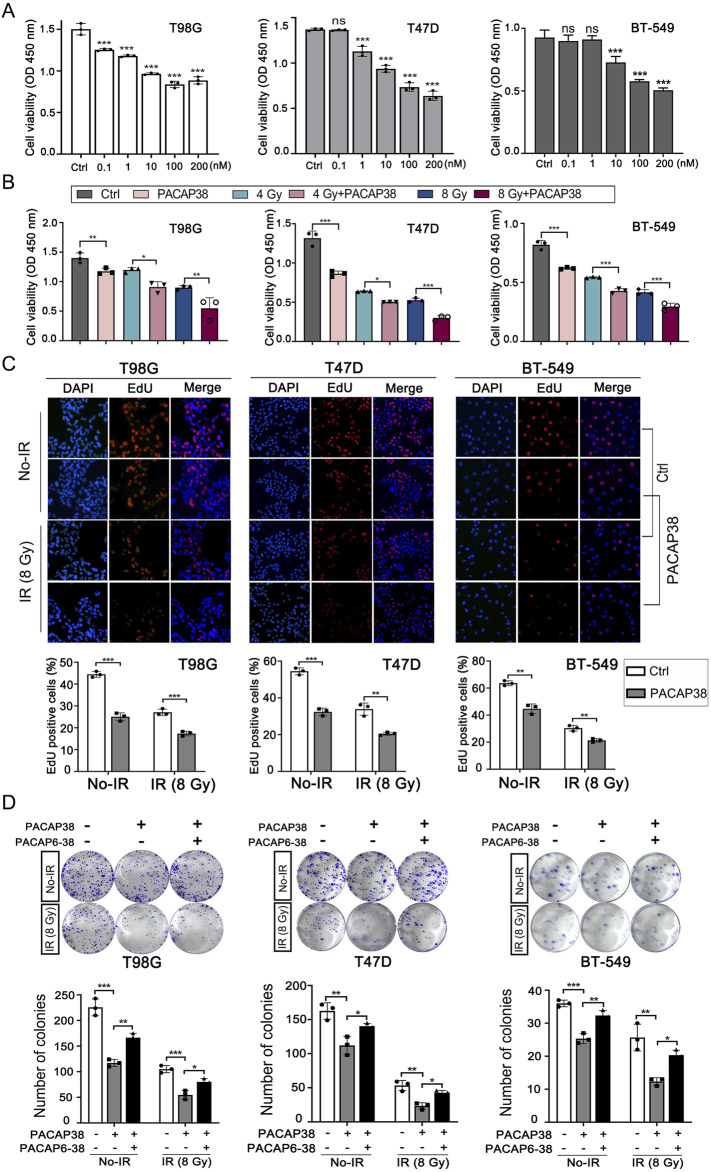
Pituitary adenylate cyclase-activating polypeptide 38 (PACAP38) synergizes with irradiation to suppress glioma and breast cancer cell proliferation. **(A)** Dose-dependent inhibitory impacts of PACAP38 on the proliferation ability of T98G, T47D, and BT-549 cells. **(B)** PACAP38 (100 nM) alone and in combination with irradiation (4 Gy, 8 Gy) could suppress T98G, T47D, and BT-549 cell proliferation *in vitro* assessed by CCK8 assays. **(C)** PACAP38 (100 nM) alone and in combination with irradiation (4 Gy, 8 Gy) could suppress T98G, T47D, and BT-549 cell proliferation *in vitro* assessed by Edu assays. **(D)** PACAP38 (100 nM) alone and in combination with irradiation (8 Gy) could inhibit T98G, T47D, and BT-549 cells proliferation *in vitro* assessed by colony formation assays. The effects were attenuated by PACAP6-38 (1 μM). Data are represented as mean ± standard deviation. *p < 0.05, **p < 0.01 and ***p < 0.001.

Based on the application concentration range of PACAP38 in previous research, we first explored how PACAP38 treatment affected the growth activity of cancer cells across a concentration range from 0.1 nM to 200 nM by CCK8 assays (Maugeri et al., 2016; Hajdú et al., 2021; Bensalma et al., 2019). The results indicated a substantial suppression of glioma and breast cancer cells (T98G, T47D, BT-549) proliferation by PACAP38 at the dose of 0.1 nM–200 nM (Figure 1A). What’s more, the dose-dependent property of this inhibitory activity is most pronounced within the range of 10–100 nM. We chose an effective dose of 100 nM in the subsequent cellular experiments *in vitro*.

We further explored the impact of PACAP38 (100 nM), applied independently and in combination with irradiation, on glioma and breast cancer cells proliferation ability by CCK8 assay and Edu assay, the colony formation capability by colony formation assay, and cell cycle by flow cytometry. The results indicated that PACAP38, in synergy with irradiation, reduced the proliferation activity (Figures 1B, C) and colony formation capability (Figure 1D) of the glioma and breast cancer cells more effective than PACAP38 was applied independently. When PACAP receptors antagonist PACAP6-38 (1 μM) was applied simultaneously with PACAP38, the suppression of colony formation capability of PACAP38 on these cancer cells was attenuated (Figure 1D). Flow experiments showed that PACAP38 increased the proportion of cells arrested in the G2-M phase that was blocked, and a higher proportion of cells were arrested in the G2-M phase when PACAP38 was co-irradiated, whereas PACAP6-38 reversed this effect of PACAP38 (Supplementary Figure S1).

### 3.2 PACAP38 suppresses tumor proliferation in a T47D-tumor-bearing xenograft mice model

**FIGURE 2:**
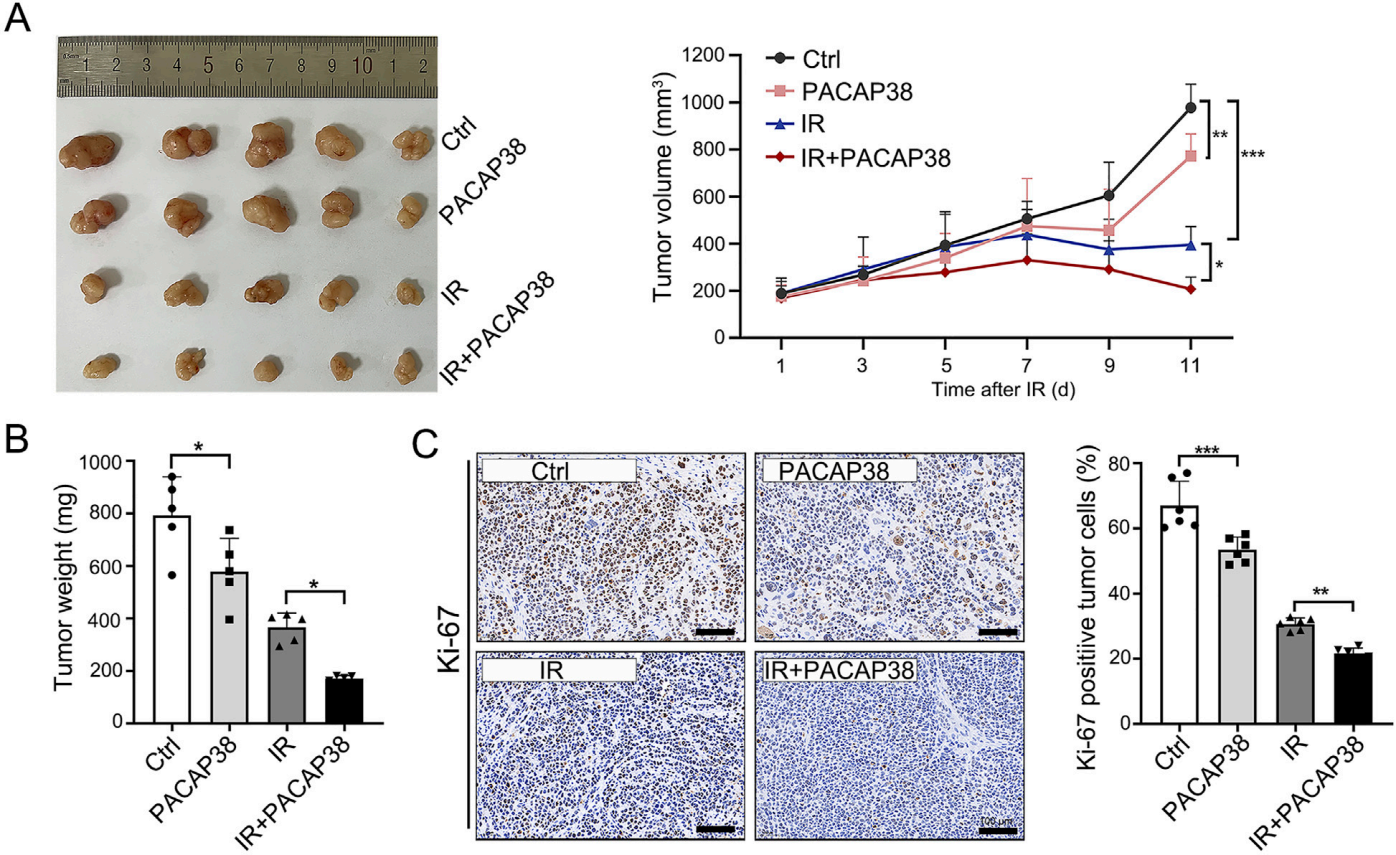
PACAP38 suppresses tumor proliferation in a xenograft tumor bearing mice model. **(A)** Images of tumors dissected from mice on day 11 post-irradiation and tumor volume records of each group (Ctrl, PACAP38, IR, PACAP38+IR) examined every 2 days from the day of IR. **(B)** The measurement and statistics of the weight of tumor tissues dissected from mice. **(C)** The Ki-67 levels in the tumor tissues dissected from mice were examined by IHC and statistically analyzed. Data are represented as mean ± standard deviation. *p < 0.05, **p < 0.01 and ***p < 0.001.

To study the impact of PACAP38 on tumor growth *in vivo*, we established a xenograft model by subcutaneously implanting T47D cells into mice. We observed substantial tumor suppression in mice that received PACAP38, irradiation, and PACAP38 combined with irradiation treatment (Figures 2A, B). Furthermore, the combination of PACAP38 and irradiation suppressed tumor proliferation *in vivo* more effective than administered with PACAP38 or irradiation alone (Figures 2A,B). The percentage of Ki-67-positive cells in dissected tumor specimens was utilized to measure the proliferation index. The IHC analyses indicated a decreased Ki-67 percentage in the PACAP38 group compared to the Ctrl group, and in the IR + PACAP38 group compared to the IR group (Figure 2C).

### 3.3 PACAP upregulated SOX6 gene expression in glioma and breast cancer cells

**FIGURE 3:**
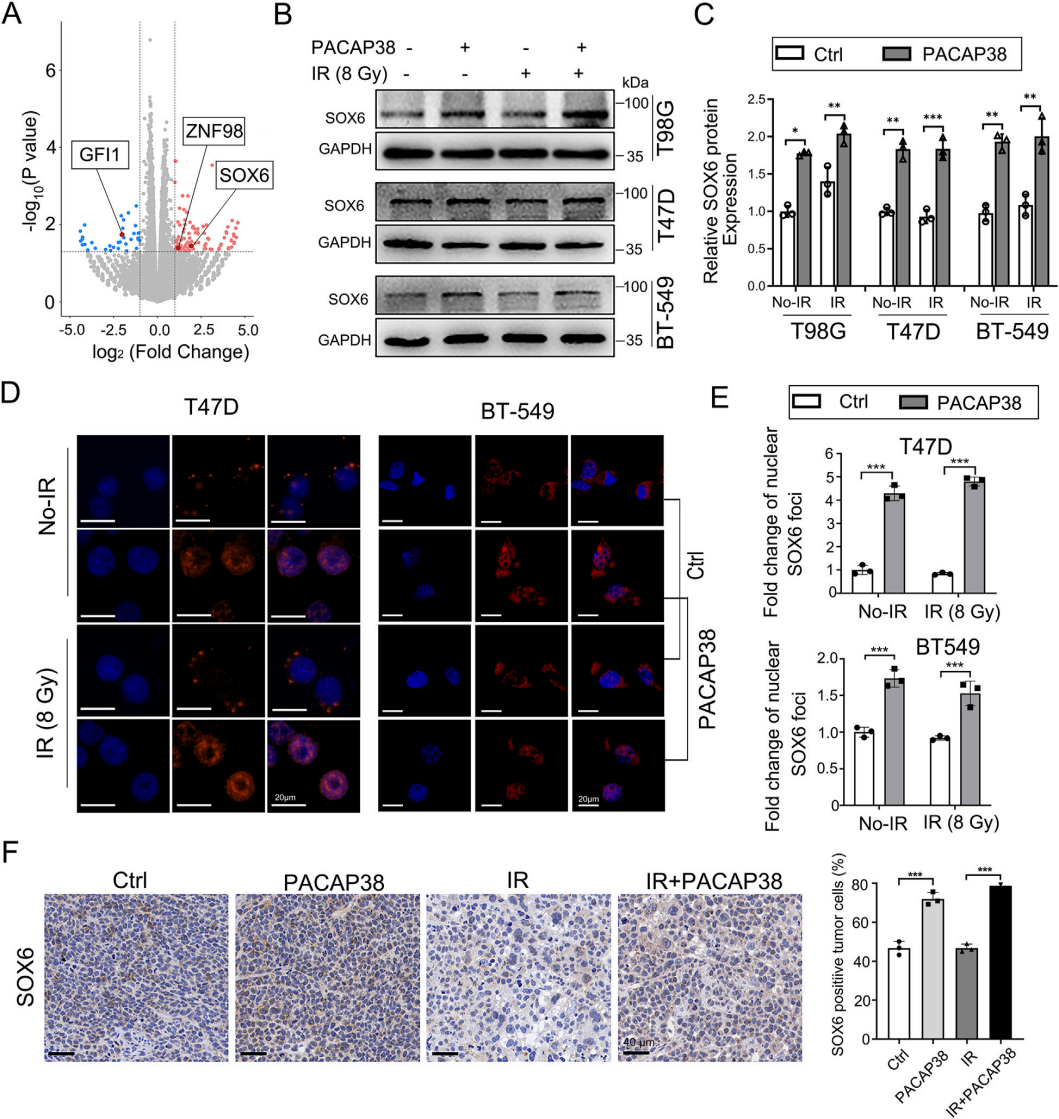
PACAP38 upregulated SRY-related high-mobility group 6 (SOX6) expression in glioma and breast cancer cells. **(A)** A volcano plot depicting all the differentially expressed genes (DEGs) in RNA samples from T47D cells treated with PACAP38 compared to those without PACAP38 treatment. Three of these DEGs (SOX6, GFI1, ZNF98), marked in the figure, belong to the transcription factor family. **(B, C)** Western blotting were performed to test the SOX6 protein expression variance between GBM and breast cancer cells treated with and without PACAP38. **(D, E)** Immunofluorescence analysis was performed to investigate the SOX6 protein localization in breast cancer cells treated with and without PACAP38. **(F)** The SOX6 protein expression levels in the tumor tissues dissected from mice were examined by IHC statistically analyzed.

To further investigate how PACAP38 suppress the growth of tumors, we conducted RNA-sequencing (RNA-seq) analysis to investigate transcriptional divergence between T47D cells treated with or without PACAP38. The transcriptional variances between the two groups revealed 174 significant differentially expressed genes (DEGs, cutoff point: |log2 fold change| > 1; *p*-value <0.05), of which 137 genes exhibited upregulation and 37 genes demonstrated downregulation in the PACAP38-treated group (Figure 3A). The information on all differentially expressed genes is presented in Supplementary Table S2. Three of these DEGs belong to the transcription factor family, with two genes (SOX6, ZNF98) upregulated and GFI1 downregulated after PACAP38 treatment.

We further validated that the level of SOX6 protein was also upregulated in PACAP38-treated T98G, T47D, BT-549 cells by western blotting (Figures 3B, C). Moreover, IF analysis showed that PACAP38 treatment enhanced the localization of the SOX6 protein in nuclear, where serving as the site for transcription factors to regulate downstream gene expression (Figures 3D, E).Immunohistochemical analysis from the preceding animal experiments indicates that PACAP38 treatment enhances SOX6 expression in tumor tissues across different groups (Figure 3F).

### 3.4 SOX6 expression correlates with tumor prognosis

**FIGURE 4:**
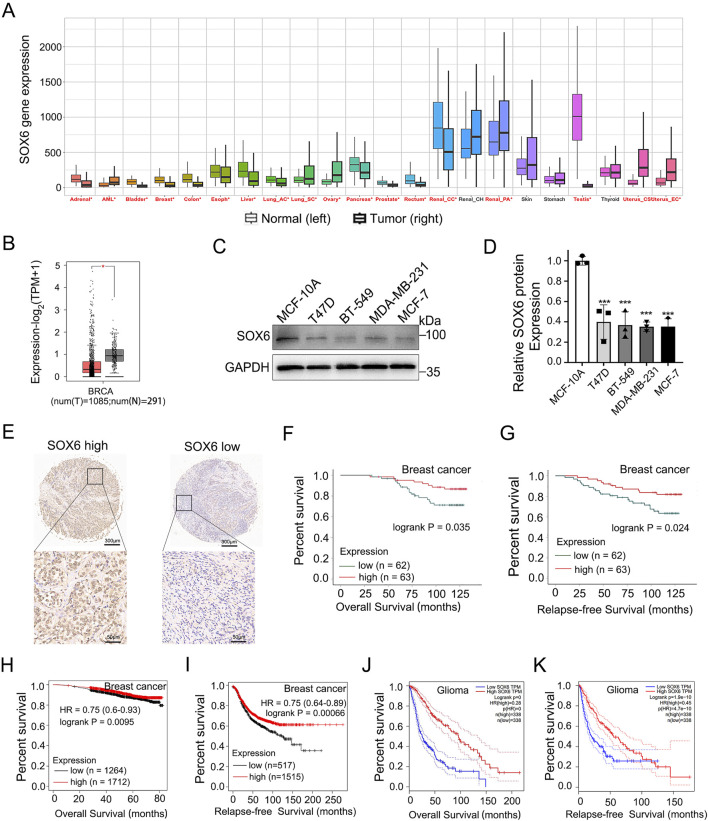
SOX6 expression correlates with tumor prognosis. **(A)** Comparison of SOX6 mRNA expression levels in pan cancerous and adjacent normal tissues via Kaplan-Meier Plotter datasets based on TCGA, GEO, EGA database. **(B)**Expression of SOX6 mRNA in 1085 tumor tissues (T) and 291 normal tissues (N) based on GEPIA database in patients with breast cancer. **(C, D)** SOX6 protein expression in human normal breast epithelial cells and several human breast cancer cell lines were detected by Western blot assay. **(E)** The SOX6 protein expression levels in the breast cancer tissue microarray obtained from Shanghai Outdo Biotech were shown. **(F, G)** Overall survival and relapse-free survival of breast cancer patients with high (n = 62) vs. low (n = 63) protein expression levels of SOX6 based on the breast cancer tissue microarray obtained from Shanghai Outdo Biotech. **(H, I)** Overall survival and relapse-free survival of breast cancer patients with high vs. low mRNA expression levels of SOX6 (auto select best cut off), as determined by Kaplan-Meier survival curve via Kaplan-Meier Plotter datasets (http://kmplot.com/) based on the TCGA, GEO, and EGA RNA databases. **(J, K)** Overall survival and relapse-free survival of glioma patients with high vs. low mRNA expression levels of SOX6 (with the median as cutoff), as determined by Kaplan-Meier survival curve via the GEPIA platform (http://gepia.cancer-pku.cn/) based on the TCGA databases. Data are represented as mean ± standard deviation. *p < 0.05, **p < 0.01 and ***p < 0.001.

Pan-cancer analysis via Kaplan-Meier Plotter datasets (http://kmplot.com/) based on TCGA, GEO, and EGA databases also indicated higher SOX6 mRNA expression in adjacent cancer tissue (normal) compared with the corresponding cancerous tissues (tumor) (Figure 4A). The SOX6 mRNA levels comparison between human breast cancer tissue and normal tissue by means of the GEPIA platform (http://gepia.cancer-pku.cn/), which is based on the TCGA and GTEx databases indicated that the levels of SOX6 expression were elevated in normal tissues (N) than in tumor tissues (T) (Figure 4B). Next, SOX6 protein levels were detected in a normal human breast epithelial cell line (MCF-10A) and several human breast cancer cell lines (T47D, BT-549, MCF-7, MDA-MB-231). The findings revealed a higher expression of SOX6 protein in normal breast epithelial cells contrast with breast cancer cell lines (Figures 4C, D).

Employing the breast cancer tissue microarray from Shanghai Outdo Biotech, a correlation analysis was conducted on the immunohistochemical expression of SOX6 in relation to clinical prognosis. The results demonstrate that high expression levels of SOX6 are indicative of improved overall survival (OS) and relapse-free survival (RFS) outcomes (Figures 4E–G). The characteristics of the clinical and pathological information of the patients involved in the chip and the correlation between the clinical and pathological characteristics of the patients and SOX6 expression were showed in the Supplementary Material (Supplementary Table S3). The results showed that there was no significant correlation between SOX6 expression and age, pathological grade, tumour size, lymph node metastasis, clinical stage and molecular type. Kaplan-Meier (KM) survival analysis of breast cancer patients via Kaplan-Meier Plotter datasets based on the TCGA, GEO, and EGA RNA databases revealed that patients with higher SOX6 mRNA levels owned an improved OS and RFS than those with lower SOX6 mRNA levels, regardless of the breast cancer subtype (Figures 4H, I). KM analysis of the GEPIA platform based on the TCGA database showed that those with higher levels of SOX6 mRNA among glioma patients had better OS and RFS than those with lower levels of SOX6 mRNA (Figures 4J, K). These results supported the SOX6 gene’s tumor-suppressive role in glioma and breast carcinoma. Based on this research, we reason that PACAP38 may suppress cancer proliferation via upregulating SOX6 expression.

### 3.5 The knockdown of SOX6 impairs the suppressive impact of PACAP38 on the proliferation ability of cancer cells

**FIGURE 5:**
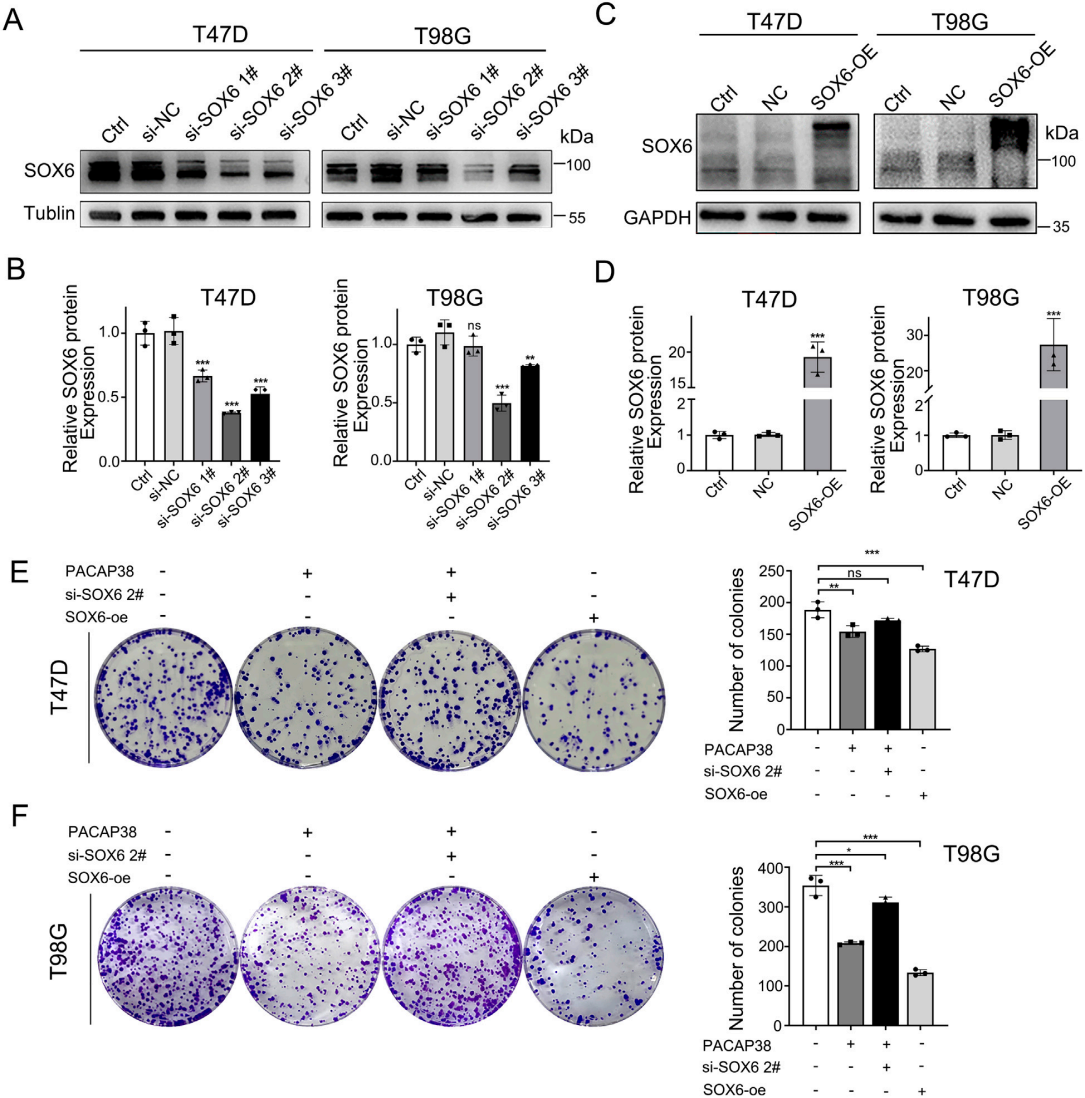
SOX6 is essential to the inhibitory effect of PACAP38 on cancer cell proliferation. **(A,B)** siRNA–mediated knockdown of SOX6 in T47D, T98G cells was evaluated by Western blotting. **(C,D)** Lentiviral mediated overexpression of SOX6 in T47D, T98G cells was evaluated by Western blotting. **(E,F)** SOX6 knockdown attenuated the ability of PACAP38 (100 nM) to inhibit colony formation capability in glioma and breast cancer cells (T98G, T47D).SOX6 overexpression in glioma and breast cancer cells inhibits the colony formation capability of cells. Data are represented as mean ± standard deviation. *p < 0.05, **p < 0.01 and ***p < 0.001.

To elucidate the role of SOX6 in PACAP38-mediated tumor suppression, we employed siRNA-mediated knockdown and lentiviral overexpression of SOX6 in tumor cell lines in T47D and T98G cells. The knockdown (Figures 5A, B) and overexpression (Figures 5C, D) efficiency were assessed by Western blot assays. Three different pairs of siRNAs sequences were used, and si-SOX6 2# was chosen as the most effective pair for further work. Colony formation assays indicated that knockdown of SOX6 could reverse the suppression of PACAP38 on T47D and T98G cells proliferation (Figures 5E, F), which indicated that the capacity of PACAP38 to suppress cancer cells proliferation is dependent upon upregulating SOX6. Additionally, the reduced proliferative capacity of tumor cells following SOX6 overexpression further validates its tumor-suppressive function.

### 3.6 The suppressive effect of PACAP38 on Wnt-β-catenin signaling is mediated by upregulating SOX6 through histone acetylation

**FIGURE 6:**
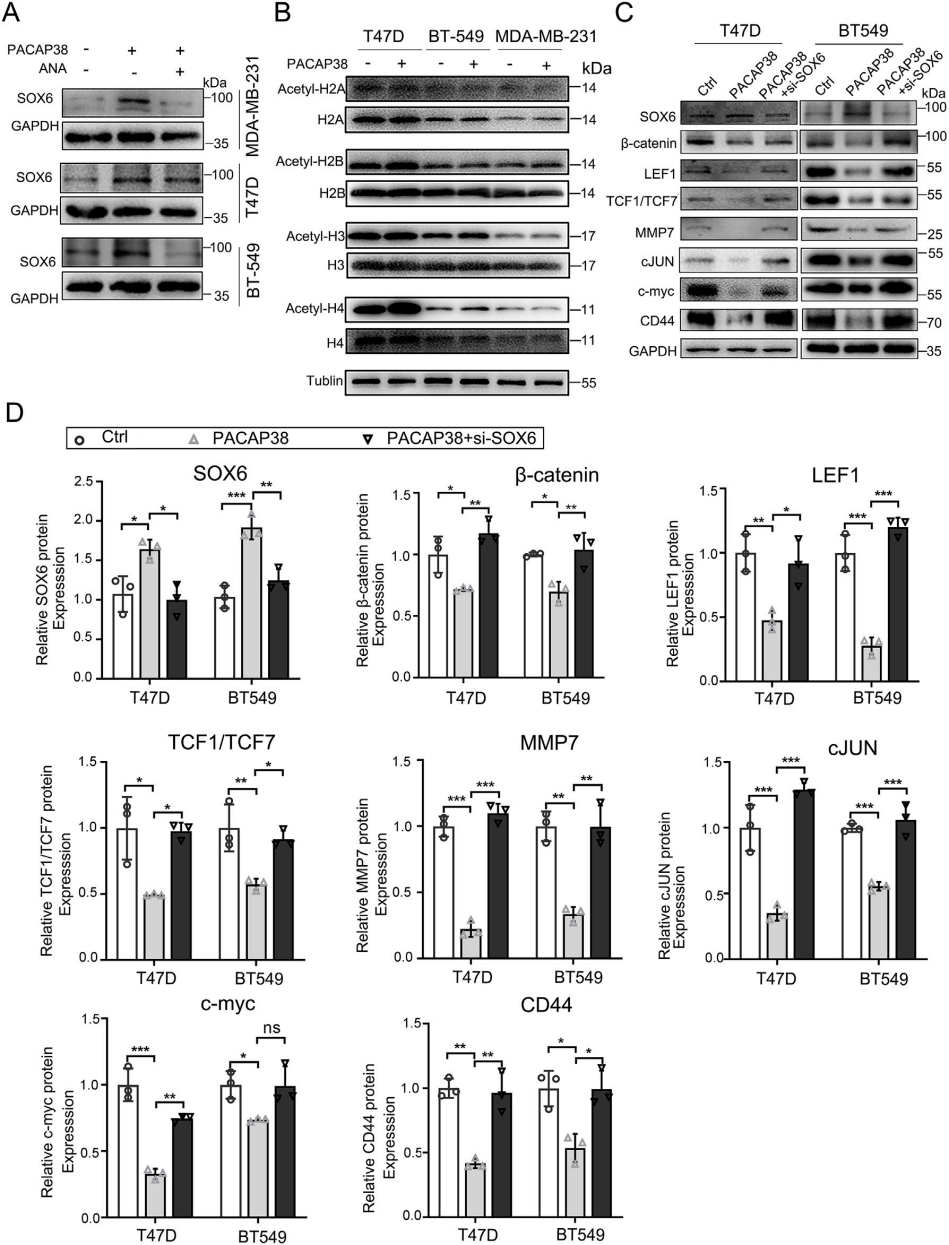
Mechanism and downstream molecular changes involved in the regulation of SOX6 by PACAP38. **(A)** Effect of ANA on PACAP38 upregulation of SOX6 expression in breast cancer cell lines were detected by Western Blotting. **(B)** Effect of PACAP on histone acetylation in breast cancer cell lines were detected by Western Blotting. **(C, D)** The level of proteins related to the Wnt/β-catenin pathway in breast cancer cells (T47D, BT549) treated with PACAP38 alone or PACAP38 in combination with si-SOX6 were assessed by Western blotting and statistically analyzed. Data are represented as mean ± standard deviation. *p < 0.05, **p < 0.01 and ***p < 0.001.

Next, we investigated the mechanisms through which PACAP38 regulates SOX6 gene expression. Epigenetic modifications are currently recognized as significant factors in the regulation of tumor pathology. We examined whether PACAP38 influences the epigenetic modifications of tumor cells at both the pre-transcriptional and post-transcriptional levels. Our findings revealed that the addition of the DNA methylation inhibitor (5-Aza) did not affect SOX6 expression when used alongside PACAP38 treatment (Supplementary Figure S2). However, the application of a HAT inhibitor (ANA) in conjunction with PACAP38 reduced the upregulation of SOX6 expression (Figure 6A). These results suggest that PACAP38 may regulate SOX6 expression through histone acetylation. We further assessed changes in histone acetylation levels following PACAP38 treatment. The results demonstrated that PACAP38 upregulated multiple types of histone acetylation including H2B, H3, and H4, with the most pronounced effects observed on histones H3 and H4 (Figure 6B). Collectively, these findings indicate that PACAP38 likely regulates SOX6 gene expression via histone acetylation.

We further explored the tumor suppression mechanisms operating downstream of the PACAP38/SOX6 axis in cancer cells. Our previous experiments indicated that PACAP38 exerted its antineoplastic effects by upregulating SOX6 expression. Wnt/β-catenin is a common downstream target pathway of SOX6. We speculated that PACAP38 may suppress Wnt/β-catenin signaling by upregulating SOX6 expression. Thus, we performed western blot assays in T47D and BT-549 cells to elucidate the mechanism responsible for the anti-cancer effects mediated by the PACAP38/SOX6 axis. Hereon, our research revealed that PACAP38 downregulated the crucial effector (β-catenin), nuclear transcription factors (LEF1, TCF1/TCF7), and downstream target genes (MMP7, cJUN, c-myc and CD44) associated with the Wnt/β-catenin signaling in cancer cells. However, when PACAP38 was applied concurrently with the knockdown of the SOX6 gene in cancer cells, the inhibitory effects of PACAP38 on proteins associated with the Wnt/β-catenin pathway were reversed (Figures 6C,D).

## 4 Discussion

**FIGURE 7:**
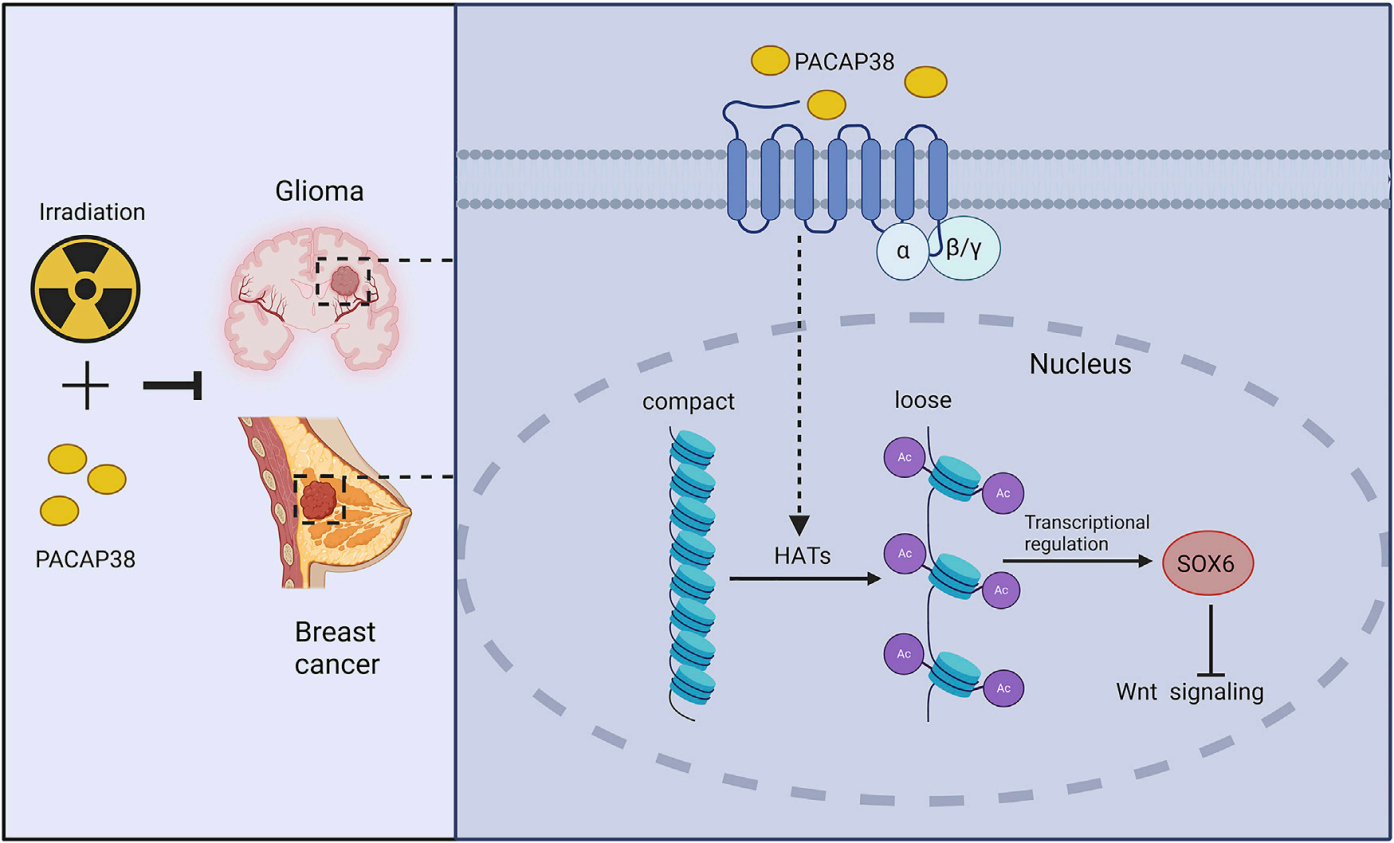
Schematic model of potential molecular pathways regulated by PACAP38 in cancer cells. PACAP38 upregulates SOX6 protein expression by prompting histone acetylation. Functioning as a transcription factor, SOX6 inhibits the expression of genes associated with the Wnt signaling. PACAP38 synergizes with irradiation to suppress the proliferation of glioma and breast cancer cell.

Our results unveiled a previously unidentified function of PACAP38 in suppressing glioma and breast cancer cells proliferation when applied alone and combined with irradiation. The suppressive effect was mediated by upregulating SOX6 gene expression and then inhibiting the Wnt-β-catenin pathway. Additionally, in this study, we are the first to discover that PACAP38 may regulate the expression of downstream proteins through histone acetylation Figure 7. Overall, the research renders PACAP38 as a novel strategy aimed at augmenting the effectiveness of radiotherapy for glioma and breast cancer.

Prior researches demonstrated the potential of PACAP38 in preventing and mitigating myocardial damage caused by irradiation (Li et al., 2019). Radiation-induced myocardial injury is a common side effect of breast cancer radiotherapy. This characteristic of PACAP38 sheds light on alleviating myocardial dysfunction caused by irradiation. While PACAP is a multifunctional peptide molecule. Most studies demonstrated that PACAP exhibits inhibitory effects on cancer (Nemetz et al., 2008; Cohen et al., 2010; Sokolowska and Nowak, 2008). The role of PACAP38 in breast cancer remains obscure. Hence, investigating the potential effects of PACAP38 on cancer cells is essential, as any promotion of cancer proliferation could potentially compromise the benefits of mitigating radiation-induced myocardial injury.

Our findings indicated that the application of PACAP38 didn’t pose a risk of promoting cancer growth. Instead, PACAP38 can inhibit cancer proliferation. Furthermore, our findings were supported by another study, which indicated that PACAP38 exhibits dual actions in multiple myeloma: it could both attenuate the injury inflicted by multiple myeloma-derived light chains on renal tubular epithelial cells and inhibit the growth of multiple myeloma cells (Li et al., 2006; Arimura et al., 2006). This differential role of PACAP in damage repair and anti-inflammatory effects in normal tissues and tumour suppression in cancer tissues may also be dependent on biological differences between normal tissues and cancer. These studies demonstrated the functional diversity of PACAP38.

Combining radiotherapy with drugs to enhance anti-tumor efficacy has become a growing trend, with approaches such as radiotherapy combined with chemotherapy, immunotherapy, and targeted therapy (Zhai et al., 2022; Meattini et al., 2024; Beddok et al., 2021). However, the variability in individual response remains for existing combination therapy strategies due to concerns about drug safety, drug-radiotherapy interactions, and the associated risk of adverse effects. Consequently, further research is needed to explore novel combination strategies and identify potential new therapeutic agents. Our study provides a promising alternative option for combining with radiotherapy in anti-tumor therapy.

PACAP38 exerts its function by binding to and activating three G-protein-coupled receptors of PAC1, VPAC1 and VPAC2, thereby activating downstream signal cascades (Harmar et al., 2012).The diversity in the receptor’s expression across different cells and the variations in PAC1 isoform may account for PACAP’s functional diversity among different cells (Dickson and Finlayson, 2009; Blechman and Levkowitz, 2013). Although the significance of distinct PAC1 splice variants under normal physiological/pathophysiological conditions remains unclear to date, the functional diversity of PACAP also relies on the interactions between its specific domains and its distinct ligands.

Our research also indicated that PACAP38 administration lead to more breast cancer cells arrest in G2/M phase. As radiosensitivity varies from different cell cycles and the G2/M phase cells own the highest sensitivity to radiation (Jeong et al., 2017), these results also suggested the potential that PACAP38 may enhance the radiosensitivity of breast cancer cells by increasing cells arrested in G2/M phase.

Past research has shown that PACAP38 exhibits suppressive effects on the growth of diverse cancer cells, but few have explored the underlying molecular mechanisms. Our research delved into the molecular mechanisms through which PACAP38 exerts its anti-cancer effects, shedding light on new directions for understanding how PACAP38 operates in the context of cancer. Past research has reported that PACAP play an important role in cartilage diseases by regulating SOX family member molecules such as SOX5, SOX6, SOX9 (Reglodi et al., 2018; Szegeczki et al., 2020; Szegeczki et al., 2019). SOX6, belonging to the SOX family, has shown its anti-tumor properties in various tumors including breast cancer (Szegeczki et al., 2019; Wang et al., 2016; Zhang et al., 2021). Currently, no studies have reported whether PACAP38 might influence the initiation and advancement of cancer by regulating SOX6. In this research, we utilized transcriptomics approaches to further probe into the mechanisms underlying PACAP38’s inhibition impact on cancer proliferation. Our research revealed that PACAP38 exerted its anti-cancer effects by upmodulating SOX6 expression. Furthermore, our studies indicated that PACAP38 synergized with irradiation *in vitro* and *in vivo* to promote the antitumor efficacy.

High expression levels of the PACAP receptors are observed across diverse cancer cell types, including breast cancer cells. This property enables them to be targeted for tumor localization imaging and antibody-based cancer therapies (Reubi et al., 2000; Moody et al., 1998; Jagoda et al., 2002; Thakur et al., 2004; Onyüksel et al., 2009; Ortner et al., 2010; Galoian and Patel, 2017). Therefore, PACAP and its receptors have the potential to be extensively applied in the tumor field. The suppressive impact of PACAP38 on cancer found in this research provides further support for the safe utilization of PACAP and its receptors in breast cancer tumor localization imaging and antibody-targeted therapies. Additionally, as an inherently safe neuropeptide, PACAP38 has not been documented to exert side effects when administered to humans at doses that result in substantial biological effects (Chiodera et al., 1996). This ensures the safety of clinical applications of PACAP38, especially in patients who require combined radiotherapy treatment. PACAP38’s role in promoting tissue repair and reducing inflammatory responses may help patients recover faster and reduce complications after cancer radiation therapy. This property makes PACAP38 not only has potential for application in anti-tumour therapy but may also play an important role in damage repair after radiotherapy.

Although our research has identified the potential of PACAP38 as a novel approach for inhibiting the proliferation and enhancing the irradiation efficiency in multiple cancer cells, numerous issues need to be addressed before its pharmaceutical application. Firstly, although PACAP38, as a natural peptide, possesses the characteristic of safety, high selectivity, and potency, the receptors for PACAP38 are broadly expressed throughout the body, particularly in the nervous system (Vaudry et al., 2009; Bourgault et al., 2008). Therefore, the toxicological effects of systemic PACAP38 administration on various aspects of mice must be further assessed. Furthermore, as a 38 amino acid polypeptide, PACAP38 exhibits some degree of instability, a short half-life, and issues related to rapid degradation (Fosgerau and Hoffmann, 2015). To develop PACAP38 into a clinical therapeutic for cancer treatment, these issues require further investigation and optimization.

## 5 Conclusion

In conclusion, this research suggests PACAP38’s potential as a promising strategy in suppressing cancer cell proliferation and enhancing irradiation efficacy. This effect of PACAP38 is likely mediated via the PKA/SOX6/Wnt-β-catenin cascade. The results of our study are promising and should encourage further preclinical and clinical investigations into the efficacy of PACAP38 for the treatment of cancer.

## Data Availability

The data presented in the study are deposited in the NCBI Sequence Read Archive (SRA) repository, accession number PRJNA1170264. Further inquiries can be directed to the corresponding authors.

## Supplementary material

SUPPLEMENTARY FIGURE S1 PACAP38 synergized with irradiation to block breast cancer cell in the G2-M phase. **(A, B)** PACAP38 (100 nM) alone and in combination with irradiation (8 Gy) could increase the proportion of T47D, and BT-549 cells arrested in G2-M phase assessed by flow cytometry. The effects were attenuated by PACAP6-38 (1 μM). Data are represented as mean ± standard deviation. **p* < 0.05, ***p* < 0.01 and ****p* < 0.001.

SUPPLEMENTARY FIGURE S2 The effects of epigenetic-modifying agents combined with PACAP38 on SOX6 expression. The effects of epigenetic-modifying aggents(5-Aza, ANA, SAHA) combined with PACAP38 on SOX6 expression in breast cancer cell.
