# Supplementary material for: PACAP38 synergizes with irradiation to suppress the proliferation of multiple cancer cells via regulating SOX6/Wnt/β-catenin signaling
Source: Front Pharmacol. 2024 Oct 22;15:1492453. doi: 10.3389/fphar.2024.1492453 (PMC11605515; doi:10.3389/fphar.2024.1492453)
Supplement: Supplementary file 2 [file Table1.docx]

Supplementary Tables

**Table S1. siRNAs sequences**

| **Gene** | **Primers** | Sense (5'-3') | Antisense (5'-3') |
| --- | --- | --- | --- |
| SOX6 | siRNA1 | GAAGCAACCUUAUUAUGAATT | UUCAUAAUAAGGUUGCUUCTT |
|  | siRNA2 | GCAAGAACAGAUUGCGAGATT | UCUCGCAAUCUGUUCUUGCTT |
|  | siRNA3 | GUACAGUUCAUUCCAUCAATT | UUGAUGGAAUGAACUGUACTT |
