# Supplementary figures and images for: PACAP38 synergizes with irradiation to suppress the proliferation of multiple cancer cells via regulating SOX6/Wnt/β-catenin signaling
Source: Front Pharmacol. 2024 Oct 22;15:1492453. doi: 10.3389/fphar.2024.1492453 (PMC11605515; doi:10.3389/fphar.2024.1492453)

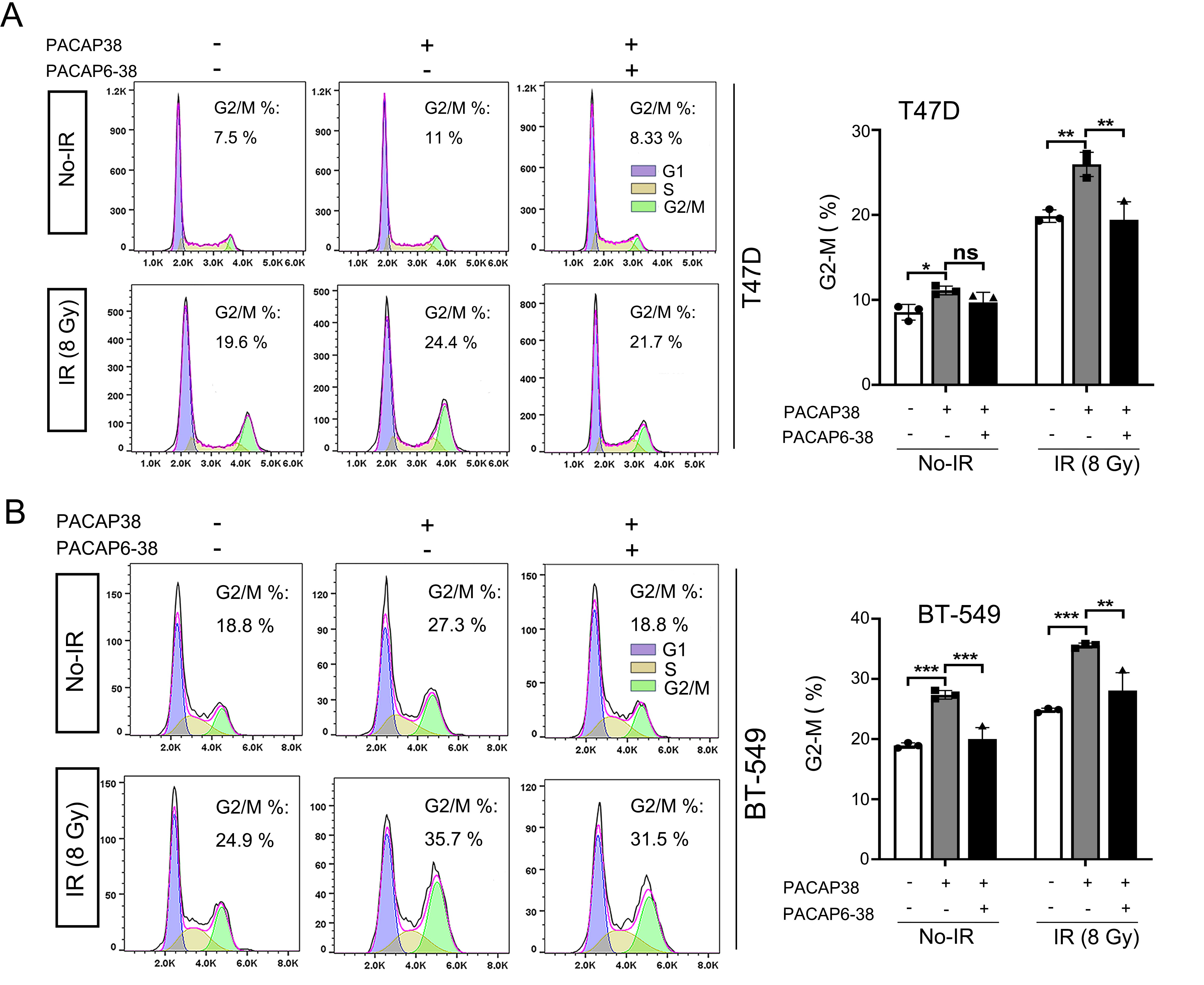

Supplement: Supplementary file 3 [file Image1.JPEG]

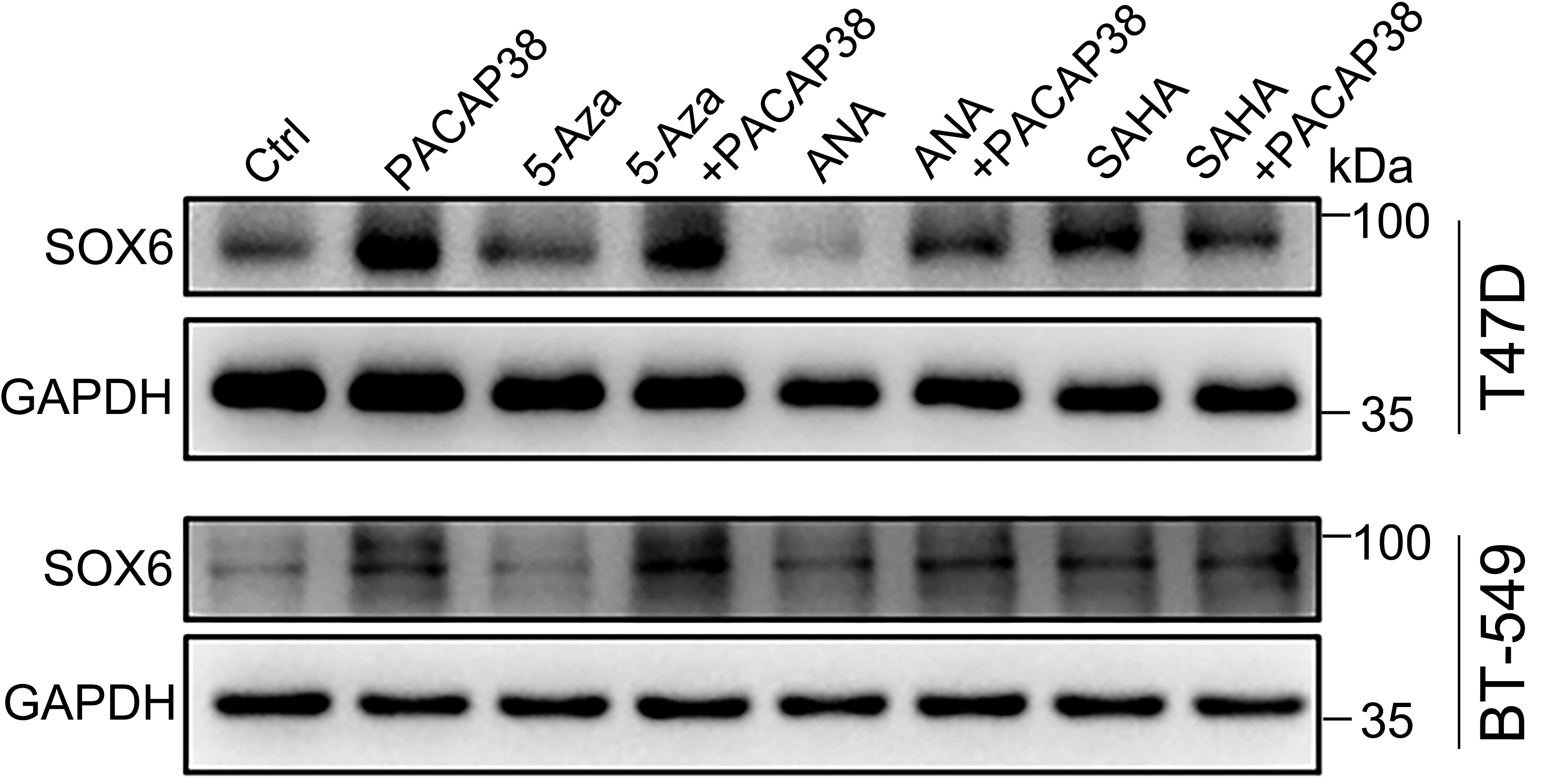

Supplement: Supplementary file 4 [file Image2.JPEG]
