# Supplementary material for: PACAP38 synergizes with irradiation to suppress the proliferation of multiple cancer cells via regulating SOX6/Wnt/β-catenin signaling
Source: Front Pharmacol. 2024 Oct 22;15:1492453. doi: 10.3389/fphar.2024.1492453 (PMC11605515; doi:10.3389/fphar.2024.1492453)
Supplement: Supplementary file 5 [file Table3.docx]

| Table S3. The relationship between SOX6 expression levels and the clinicopathological characteristics of breast cancer. | | | | |
| --- | --- | --- | --- | --- |
| Total（N=125） | | SOX6 expression levels | | *p* value |
|  |  | low | high |  |
| **Age(years)** |  |  |  | 0.853 |
| ≤50 | 45 | 23 | 22 |  |
| ＞50 | 80 | 39 | 41 |  |
| **Pathological grade** |  |  |  | 0.08 |
| Grade II | 87 | 48 | 39 |  |
| Grade III | 38 | 14 | 24 |  |
| **Tumor stage** |  |  |  | 0.713 |
| T1 | 47 | 22 | 25 |  |
| T2-3 | 78 | 40 | 38 |  |
| **Nodal stage** |  |  |  |  |
| N0 | 65 | 33 | 32 | 0.858 |
| N1 | 26 | 11 | 15 |  |
| N2 | 28 | 15 | 13 |  |
| N3 | 6 | 3 | 3 |  |
| **AJCC** **stage** |  |  |  | 0.113 |
| Stage I | 26 | 10 | 16 |  |
| Stage 2 | 57 | 34 | 23 |  |
| Stage 3 | 42 | 18 | 24 |  |
| **Molecular subtype** |  |  |  | 0.457 |
| Her-2 | 9 | 5 | 4 |  |
| Luminal | 91 | 43 | 48 |  |
| TNBC | 21 | 13 | 8 |  |
| **Her-2 status** |  |  |  | 0.814 |
| Negative | 99 | 50 | 49 |  |
| Positive | 22 | 10 | 12 |  |
| **ER status** |  |  |  | 0.23 |
| Negative | 35 | 21 | 14 |  |
| Positive | 86 | 40 | 46 |  |
| **PR status** |  |  |  | 0.717 |
| Negative | 57 | 27 | 30 |  |
| Positive | 65 | 34 | 31 |  |
